# Assessing safety climate in prehospital settings: testing psychometric properties of a common structural model in a cross-sectional and prospective study

**DOI:** 10.1186/s12913-019-4459-5

**Published:** 2019-09-18

**Authors:** Leif Inge K. Sørskår, Espen Olsen, Eirik B. Abrahamsen, Gunnar Tschudi Bondevik, Håkon B. Abrahamsen

**Affiliations:** 1 Institute for Safety, Economics and Planning, University of Stavanger, Kjølv Egelands hus, Kristine Bonnevies vei 22, 4021 Stavanger, Norway; 2 Department of Innovation, Management & Marketing, UiS Business School, University of Stavanger, Elise Ottesen-Jensens hus, Kjell Arholms gate 37, 4021 Stavanger, Norway; 3 Department of Global Public Health and Primary Care, University of Bergen, Kalfarveien 31, 5018 Bergen, Norway; 4 National Centre for Emergency Primary Health Care, NORCE Norwegian Research Centre, Kalfarveien 31, 5018 Bergen, Norway; 5 Department of Anesthesiology and Intensive Care, Stavanger University Hospital, Gerd Ragna Bloch Thorsens gate, Stavanger, 4011 Norway

**Keywords:** Prehospital, Emergency medical services, Helicopter emergency medical services, Patient safety culture, Patient safety climate, HSOPSC, Psychometric properties

## Abstract

**Background:**

Little research exists on patient safety climate in the prehospital context. The purpose of this article is to test and validate a safety climate measurement model for the prehospital environment, and to explore and develop a theoretical model measuring associations between safety climate factors and the outcome variable transitions and handoffs.

**Methods:**

A web-based survey design was utilized. An adjusted short version of the instrument Hospital Survey on Patient Safety Culture (HSOPSC) was developed into a hypothetical structural model. Three samples were obtained. Two from air ambulance workers in 2012 and 2016, with respectively 83 and 55% response rate, and the third from the ground ambulance workers in 2016, with 26% response rate. Confirmatory factor analysis (CFA) was applied to test validity and psychometric properties. Internal consistency was estimated and descriptive data analysis was performed. Structural equation modelling (SEM) was applied to assess the theoretical model developed for the prehospital setting.

**Results:**

A post-hoc modified instrument consisting of six dimensions and 17 items provided overall acceptable psychometric properties for all samples, i.e. acceptable Chronbach’s alphas (.68–.86) and construct validity (model fit values: SRMR; .026–.056, TLI; .95–.98, RMSEA; .031–.052, CFI; .96–.98). A common structural model could also be established.

**Conclusions:**

The results provided a validated instrument, the Prehospital Survey on Patient Safety Culture short version (PreHSOPSC-S), for measuring patient safety climate in a prehospital context. We also demonstrated a positive relation between safety climate dimensions from leadership to unit level, from unit to individual level, and from individual level on the outcome dimension related to transitions and handoffs. Safe patient transitions and handoffs are considered an important outcome of prehospital deliveries; hence, new theory and a validated model will constitute an important contribution to the prehospital safety climate research.

## Background

The prehospital environment is characterized by time pressure, high activity, uncertain situations, changing environments and a high dependency on the emergency medical providers’ teamwork, competence and communication abilities: a demanding mix, prone to errors. Undesirable events and near misses seems to be more recurrent within this environment than what is reported or shared [1]. Compared to the hospital context, little research exists on patient safety in the prehospital context [2–4] and on the relationship between organizational factors creating barriers and increased patient safety [5]. Safety culture is considered an important theme and premise for continued improvement of patient safety [6, 7]. There is mounting evidence on the relationship between improved safety culture and less occurrence of adverse events [8–12] and improved safety performance [13, 14].

Different from safety culture, safety climate is defined as “*surface features of the safety culture from attitudes and perceptions of individuals at a given point in time*” [15], i.e. safety climate research is viewed as a ‘snapshot’ of the safety culture [16]. A growing number of studies report on the value of safety climate assessments [14, 17]. Research also indicates a strong connection between safety climate and safety behavior [18–20], and that safety-related outcomes such as accidents may be predicted by safety climate assessments [21]. In health care such assessments are conducted to reveal, keep track of, and manage safety issues, in addition to evaluate interventions and trends [6, 18, 22]. An area viewed as critical in the eyes of the Emergency Medical Services’ (EMS) providers is transitions and handoffs, since this brief window provides the opportunity to influence the further course of their patients’ care [23]. To make improvements in transitions and handoffs, the first step is for the policy decision-makers to understand how the workers perceive their organization’s patient safety climate [24].

Survey methods are considered a good approach to perform safety climate assessments [25], and several instruments have been developed for application within health care services [15], some of which are also intended for use within acute hospital settings [26]. One such instrument is the Hospital Survey On Patient Safety Culture (HSOPSC), developed in 2004 by the Agency for Healthcare Research and Quality (AHRQ), and later translated to several languages [27]. The Norwegian version of the HSOPSC instrument was recently adjusted and validated for the prehospital environment [28]. Another instrument is a rebuilt shorter version, the HSOPSC-S, which has been validated for the hospital and the petroleum sector [29]. HSOPSC-S was developed with the help of exploratory factor analysis (EFA) to explore the possibility of a common structural model for measuring the safety climate in two different sectors: the health care sector and the petroleum sector. The HSOPSC-S is based on a multilevel theoretical framework, emphasizing that all levels in an organization have safety functions and influence performance at the individual level [29].

In the prehospital environment in Norway, car-, boat-, and air ambulances constitute the main part of the EMS activities. Although the different types of ambulances has similar goals and tasks, there are also substantial differences, such as team compositions, worker competence groups, care and treatment processes, and the physical environment [30]. This makes it valuable to provide a validated safety-climate instrument, suitable for application in these different subgroups.

Due to the fast-pace work culture in the prehospital environment, a shorter instrument seems beneficial, as it may be challenging to perform frequent assessments of the patient safety climate. There is also a focus in the Norwegian health sector on developing shorter tools for the measurement of health, safety and environment (HSE) and culture [31]. As the prehospital setting differs from the hospital setting, it is necessary to perform a new test of the psychometric properties for HSOPSC-S, preferably split into different subgroups. Such testing is beneficial, as research on psychometric properties, including the reliability and validity of replicated instruments, is continually needed [32–36]. It is also recommended to develop theoretical models to understand the safety climate impact on outcomes [26]. The aims of our study were to:

1. Test psychometric properties and validate an adjusted version of Norwegian HSOPSC-S performed on three samples from the prehospital environment: two samples from the Helicopter Emergency Medical Services (HEMS; air ambulance) retrieved at different points of time and one sample from the ground ambulance.
2. Test and further develop a theoretical model using a structured equational modelling (SEM) approach to create a fit for three prehospital samples, i.e. to test the network of relationships between the variables in a theoretical framework of HSOPSC-S in a prehospital setting.

### Theoretical background

**Fig. 1:**
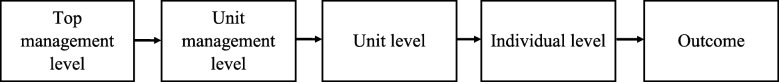
Hypothetical prehospital multilevel model

Patient safety is a broad endeavor that requires thinking beyond the individual patient to consider the characteristics of the whole system [37]. It is reasonable to consider the prehospital domain as part of the whole healthcare system, and in particular as part of the treatment chain. The importance of performing research in this domain is reflected by the limited amount of prehospital research on patient safety, compared to the amount of hospital research. Taking a multilevel perspective when performing assessments of safety climate in organizations has been suggested [18, 38]. Figure 1 is a simple illustration of a hypothetical prehospital multilevel system model, on which we build our theoretical structured framework.

#### Top and unit management levels

It is well known that leadership has a strong cultural influence on an organization through its beliefs and values [39] and that effective leadership promotes better patient outcomes [1]. Top management’s safety-related attitudes and behaviors will form the basis for healthcare providers’ safety behavior and hence the organization’s safety performance [1]. Middle management, i.e. unit management, is responsible for transforming priorities and values into operating procedures and action guidelines [40] and the view that top management’s influence on unit management supervisory practices is aligned with a multilevel perspective on safety climate [41, 42]. Supervisors achieve higher levels of safety compliance from their subordinates if the subordinates perceive that the supervisors prioritize safety [43]. The decentralized nature of the prehospital setting, relative to the hospital setting, is an important aspect in this matter. One might speculate that prehospital unit management might have a more important role, compared to that of their hospital peers, in their influence on the integration of systems, patient safety interventions and staff performance. With the help of coaching, mentoring and the fostering of mutual respect, staff may incorporate management’s priorities and values into their daily work, helping them to develop their own leadership potential [7].

Different leadership styles seem to influence safety-relevant behavior [41]. Several studies suggest that there is a correlation between transformational leadership and organizational outcomes [43–45]. Transformational leadership can also function as an antecedent of climate strength [46]. Transformational leadership is conducted on organizational workers to raise their awareness of the importance of task outcomes, to meet the team members’ needs, and to induce them to prioritize the organizational goals [47]. Effective leadership promotes a setting in which healthcare providers are treated respectfully, consequently improving their performance [1]. It is also essential that unit leadership behavior creates an atmosphere in which team members feel they can communicate openly and participate in decision-making [48] and speak up if they have safety concerns [49]. In addition, the prehospital unit management conducts leadership in the local unit by assigning teams to the different unit transport vehicles, from which the emergency dispatcher allocates the designated teams’ medical emergencies, transport assignments or similar. In the prehospital environment, critical tasks are performed in teams without direct supervision from the unit management, which implies that the leadership task of putting together teams influences team outcomes.

#### Unit and individual level

For different high-stake contexts, a close relationship between teamwork and performance has been identified [50–55]. Teamwork is generally considered to be the basis for good patient care [56], and it is also valuable within fast-paced unpredictable environments [57]. Teamwork is also regarded as of critical importance in assuring patient safety [49, 58]. Poor teamwork and communication have emerged as contributions to medical errors [50, 51]; in particular, a lack of cross-monitoring of the actions of team members has been identified as a significant contributor to teamwork failure [54].

In acute healthcare settings, teams are hierarchical [1], and team leaders are responsible for executing operational procedures and action guidelines given by the unit management [42]. On one side, the positive attribute of hierarchy is the clear path of information and decision-making in critical situations. On the other side, hierarchy may hinder problem-solving, e.g. if team members leave everything to the team leader and do not contribute to collecting data or finding the best treatment option [1]. Although hierarchy, formal or informal, applies in the majority of the prehospital setting, HEMS is an exception to this, as the team members are responsible for different domains and tasks, according to their profession.

In addition to effective leadership and teamwork, key elements of a safety culture also include learning from errors and developing an open environment based on trust [7]. A recent study identified two effective interventions improving the governance of patient safety within organizations: simulation-based training is increasingly valued as an effective method to enhance safety knowledge and behavior, and, secondly, the use of well-designed incident reporting leads to increased learning, feedback and improvement within units [59]. Both are among many activities that could be undertaken to create organizational learning.

It is common to define organizational learning as change in the organization’s knowledge that occurs as a function of experience [60]. Organizational learning is a dynamic process [61], meaning that, in addition to occurring over time and between organizational, unit and individual levels, learning creates a tension between assimilating new learning (feed forward) and using what is already learned (feedback). For the purpose of assimilating new learning in the prehospital setting, it is important that sufficient resources for effective and efficient reporting are available [59]. It is also important to strive for an open and fair culture to achieve open discussions about things that went wrong and when, how, and in what context they occurred [62]. In addition, such a culture should encourage healthcare providers to report patient safety incidents [7].

The degree of risk for the patient in a decision-making situation depends on the healthcare providers’ (a) awareness and knowledge of threats, (b) experience and practice with similar situations, and (c) actual clinical competence [1]. Individual team members’ behavior is influenced by the team they work in, i.e. how they communicate, support and supervise each other [37]. Learning is also dependent on other individuals; e.g., inexperienced workers learn by observing more experienced colleagues’ actions and related consequences [1].

#### Outcome: transitions and handoffs

Patient transitions and handoffs in hospitals have the potential for errors [24, 63], and, recently, transitions and handoffs have gained greater attention, also in the prehospital domain [5, 23, 64–67]. Due to the characteristics of the prehospital context, transitions and handoffs are both challenging and complex as they involve a number of different people, such as both hospital and prehospital staff, patients, the public, and a range of communication technologies and formats [5]. Several factors could prevent effective and high-quality transitions and handoffs, e.g. lack of understanding between health care disciplines, inattention, variable quality and quantity of information exchanged, and busy and complex situations [68]. Some poor communication practices are rooted in individual behavior, such as not listening, relational issues, or misunderstandings [5]. Heavy workload in the emergency department may induce reduced “active listening” [64, 69–71]. Errors also tend to follow unsafe behavior, e.g. when workers do not follow procedures or rush to finish tasks [18]. A recent study found it disturbing that they observed no effective interventions to monitor or improve patient safety in the prehospital chain, considering the high number of patient transitions and handoffs between EMS organizations [59].

Good transitions and handoffs are associated with improved patient safety, continuity of patient care and improved decision-making [64, 71–73] and are clearly more than an exchange of information [65]. Quality of care is dependent on the transfer of patient information, and, since verbal transfer is often only 50% accurate, a technical solution is likely to improve patients’ outcome [67]. Nevertheless, due to the inherent complexity found in healthcare settings and communication tasks, this indicates that communication issues may not be solved by technical solutions alone [5]. The quality and quantity of information has been found to correlate with the ambulance personnel’s level of formal training and experience [71, 74]. To provide quality patient transitions and handoffs from prehospital to in-hospital, it is imperative to have ongoing formal learning in interdisciplinary teamwork, communication and a structured flexible framework in a supportive work environment [68]. Four potential improvements have been identified for transitions and handoffs from prehospital to in-hospital: direct communication between the EMS provider and the physician responsible for the patient’s care; interdisciplinary communication between hospital and prehospital staff; standardization of many of the aspects of transitions and handoffs; and improved information technology [23]. There seems to be a need for a broader conceptualization of patient transitions and handoffs, including social and organizational factors, some of which are embedded in the organization’s safety culture.

A recent study was performed on whether an organization’s safety culture factors influence effective handoffs within hospitals [24], especially the effect from an organization’s communication, teamwork, reporting and management cultures. They found that these safety culture composites influence different aspects of transitions and handoffs, i.e. information, responsibility and accountability. Viewing transitions of care as a more specific variant of patient safety culture attenuates ambiguity so that the stakeholders may more easily identify and align with the goals and processes of patient safety improvement programs [24]. Based on this, we made a major adjustment to the theoretical framework that constitutes the HSOPSC-S. The safety climate dimension “*transitions and teamwork across units*” was replaced with the safety climate dimension “*transitions and handoffs*” from the original HSOPSC, and, moreover, it was treated as an outcome dimension in our model.

### Theoretical model and hypotheses

In summary, we hypothesize that the prehospital safety climate is aligned with a multilevel perspective, i.e. that the higher levels have an influence on the lower levels, as previously tested in the hospital sector and the petroleum sector [29]. Further, based on knowledge about the prehospital setting, theory and earlier research, we hereby argue that patient transitions and handovers must be considered an important outcome variable; handovers and transitions are the final ‘product’ of the prehospital chain. As it is found that an organization’s safety culture composites have an influence on patient transitions and handoffs [24], we hypothesize that all safety climate dimensions in HSOPSC-S have an influence on the outcome dimension “*transitions & handoffs*”. The theoretical, structured framework is based on the following hypotheses:

- Organizational management support for safety will enhance transitions and handoffs.
- Organizational management support for safety will enhance supervisor/manager expectations and actions promoting safety at the unit level.
- Manager expectations and actions promoting safety within units will enhance transitions and handoffs.
- Manager expectations and actions promoting safety within units will enhance learning, feedback, and improvement within units.
- Manager expectations and actions promoting safety within units will enhance teamwork within units.
- Learning, feedback, and improvement within units will enhance teamwork within units.
- Teamwork within units will enhance safety behavior.
- Learning, feedback, and improvement within units will enhance safety behavior.
- Teamwork within units will enhance transitions and handoffs.
- Learning, feedback, and improvement within units will enhance transitions and handoffs.
- Safety behavior will enhance transitions and handoffs.

**Fig. 2:**
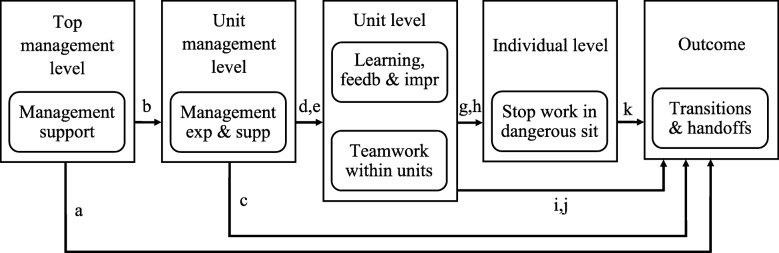
Hypothetical structured framework. Note: Management support = “*Management support for patient safety*”, Management exp. & supp = “*Manager expectations & actions promoting patient safety”*, Learning, feedb & impr = “*Learning, feedback & improvement within units*”, Stop working in dangerous sit = “*Stop working in dangerous situations*”

Hypothesis a-h are consistent with the original theoretical framework [29], and hypothesis i-k are adjustments for this study. Figure 2 displays the hypothesized theoretical, structured framework.

## Methods

### Population characteristics

In the prehospital environment in Norway, regional health trusts are responsible for the EMS activities (car-, boat-, and air ambulance). Regulations state that car ambulances should be staffed by at least two people; either two emergency medical technicians (EMT) or one EMT and another licensed health care worker with necessary EMS competence, e.g. a paramedic, a nurse or a physician [30]. For the boat ambulance, at least one person must be an EMT, in addition to the skipper. For both car- and boat ambulances, the transportation of critically ill patients may require special medical competence, often provided by accompanying healthcare personnel [30]. For the Helicopter Emergency Medical Services (HEMS; air ambulance), regulations state that the staff should consist of a physician/anesthesiologist and HEMS crew member (HCM), both with the required competences, in addition to the pilot. HEMS is a high-competence service and represents the sharp end in the prehospital chain [30]. The content of this paragraph is previously described in [28].

### The Norwegian HSOPSC-S and pre-testing of the instrument

The original Norwegian HSOPSC-S consists of six dimensions and 21 items. The response format ranges from 1 (disagree strongly) to 5 (agree strongly) on a Likert scale. There are also seven items relating to the respondents’ work characteristics (*work area, geographic location, field of competence, patient contact, work hours, seniority in the prehospital area, seniority in position*). A pre-test of the instrument’s items was performed in collaboration with prehospital professionals to ensure correct terminology in the prehospital context. Although minor adjustments had to be implemented, and some items were pointed out as challenging in the prehospital context, no items were left out or conceptually changed before distribution of the survey. The HSOPSC-S items is retrieved from [28, 29], and minor adjustments and challenging items is thoroughly discussed in [28].

### Data collection

The target group of this study consists of two main groups of employees: HEMS and ground ambulance personnel. The professional team composition of the ground ambulance is very different from that of HEMS. A HEMS team normally consists of a physician/anesthesiologist, a HCM and a pilot. One base in Norway operates with a nurse aboard, in addition to the aforementioned three-man crew. The three largest professional groups in the ground ambulance are EMTs, paramedics and nurses.

The HEMS samples were retrieved in 2012 and 2016, and the ground ambulance sample was retrieved in 2016 from questionnaires performed in 17 (of 18) health trusts.

The 2012 sample was retrieved from a survey among crew members in the civilian Norwegian HEMS. To maximize the response rate, a commentary on the upcoming study was published in the Norwegian Medical Journal. The survey was distributed via both e-mail, with a link to a web-based questionnaire (Questback), and an identical paper version along with prepaid stamped return envelopes. Data were collected between May and July 2012. All crew members received a follow-up phone call as a reminder and encouragement to answer 2–4 weeks after the survey was commenced.

For the 2016 samples we retrieved e-mail addresses for prehospital personnel in the Norwegian ground ambulance and HEMS from prehospital system leaders. We applied a web-based tool (SurveyXact) to conduct the survey, and an individual link to the questionnaire was distributed by e-mail to all personnel. Data were collected between October and December 2016, and non-responders received up to five reminders before the study was closed.

### Statistical analyses

Psychometric properties for the assessment of validation were applied [75, 76], to evaluate the HSOPSC-S for the different samples. Negatively worded items were reversed, and confirmatory factor analysis (CFA) was conducted to analyze the construct validity and to determine the degree of fit between the constructed measurement instrument and the sample. Covariation between underlying dimensions was permitted.

The following indices were applied for global fit assessment: Standardized Root Mean Square Residual (SRMR), Tucker-Lewis Index (TLI), Root Mean Square Error of Approximation (RMSEA) and Comparative Fit Index (CFI). Values for TLI and CFI in the 0.90s are generally accepted as guidance values for an acceptable fit, and those above 0.95 reflect a good model fit [77, 78]. It has been suggested using a two-index strategy by reporting SRMR with one of the fit indices (e.g. CFI or RMSEA), with the guidance criteria CFI > 0.95, SRMR < 0.8 and RMSEA < 0.6 [79]. A value below 0.5 is considered a good fit for RMSEA [77]. Guidance values should be adjusted in regard to both complexity of the model and to sample size [76]; see Table 1. Chi Square (Χ ^2^) is not reported as it has been shown to be problematic in model fit assessments for larger samples [80]. The methodology described in this paragraph is previously described in [28].

**Table 1:** Guidance values for model fit indices for models consisting of 12–30 items

Indices	*N* < 250	*N* > 250
Standardized Root Mean Square Residual (SRMR)	< .08	< .08
Tucker-Lewis Index (TLI)	> .95	> .92
Root Mean Square of Approximation (RMSEA)	< .08	< .07
Comparative Fit Index (CFI)	> .95	> .92

N = sample size. Retrieved from [76]

Modification of the instrument to gain model fit for all samples was performed with the help of modification indices and standardized residuals [75]. Values above 3.84 are statistically significant for modification indices. Standardized residual covariances for the different items should be below |4.0| and preferably below |2.5|; i.e., values above |2.5| indicate a concern with an item and values above |4.0| indicate candidates for removal [76]. In addition, items with weak loadings were considered for removal. The reader should be aware that such post hoc modifications to models should normally be done sparingly and founded on theoretical and practical plausibility [81].

To assess configural variance, the GEMS and the two HEMS samples were entered as a grouping variable when CFA was conducted for the total sample of data. Configural invariance is an approach to test measurement invariance which is related to the concept validity of measures. Configural invariance is applied to test the samples together and freely without cross-group constraints [82].

To indicate discriminant validity, MANOVA (multivariate analysis of variance; Wilks’ Lambda) was performed to investigate whether the different work characteristics had an influence on the overall statistical variance of the safety climate dimensions. To demonstrate convergent validity for a latent construct, items with high loadings on a factor should be observed, as they would indicate convergence against a common point. All item loadings for a factor should be at least 0.5 or higher (ideally 0.7 or higher) for standardized estimates, in addition to being statistically significant [76]. On the other hand, several loadings at very high levels are not desirable, as this would indicate a lack of discriminant validity for the factor items. A range of loadings between 0.6 and 0.9 seems reasonable [75]. Inter-correlation between the dimensions was examined by the Spearman-Rho correlation. Cronbach’s alphas were estimated, to determine whether factor scales yielded acceptable internal consistencies with alpha coefficients between 0.70 and 0.90 [83]. The methodology described in this paragraph is previously described in [28].

After obtaining model fit of the measurement model for all subgroup samples, the second step was to perform SEM to test the hypotheses. For this study, we applied a specified criterion that structural relations between two latent factors should be significantly valid (*p* < .05) in at least two of the three samples. This approach has been adequately demonstrated in another study [84]. Following this criterion, structural relations that did not meet this criterion were removed so that a final robust model could be established. Lastly, the final structural model was compared to a model where all direct effects was estimated. This is conducted to compare the psychometric difference between the hypothetical and alternative model.

Confirmatory factor analysis (maximum likelihood) was estimated using AMOS 25.0. The other statistical analyses were performed using SPSS 25.0.

### Ethics approval and consent to participate

For the 2012-data the Regional Committee for Medical and Health Research (REC) South-East Norway (Ref. number 2010/3326) reviewed and approved the study. Participating was voluntary, and consent was given by responding to the questionnaire. For the 2016-data approval was obtained from the Norwegian Social Science Data Services (NSD; project number 45723). All participants received information regarding the purpose of the study; they were assured that the digital questionnaires were to be treated in confidence and that no participants could be identified in the published material. Their written consent to participate in the study was given at the start of the survey.

## Results

### Response rates

The HEMS 2012 sample consisted of 172 (83% response rate) individuals participating, with 145 (70%) completed questionnaires. The HEMS 2016 sample consisted of 118 (55%) individuals participating, with 109 (51%) completed questionnaires. The Ground 2016 consisted of 1269 individuals participating (26%) with 1045 (21%) completed questionnaires.

A few responses in both HEMS samples reported their work area to be Search and Rescue Services (SAR) or Fixed Wing air ambulances (FW), but these were excluded since their mission profile and crew concepts differ substantially from HEMS.

For the analyses, only returned questionnaires with all items answered were used (listwise deletion). The majority of incomplete questionnaires was discontinued early in the survey, and we evaluated that replacing missing values was not expedient.

### Sample characteristics

The sample sizes are considered representative, based on variation in demographic variables, e.g. distribution across professional groups, range in seniority, and geographic location (Table 2).

**Table 2:** Demographic and professional characteristics of the three samples in the study (2016 samples similar to [28])

Characteristics	HEMS 12 N (%)	HEMS 16 N (%)	Ground 16 N (%)
Sample size	145	109	1045
Professional group			
EMT			544 (52)
Paramedic			260 (25)
Nurse EMT			146 (14)
Nurse	6 (4)		40 (4)
EMT Apprentice			24 (2)
Other healthcare			25 (2)
Administrative			6 (< 1)
Physician	74 (51)	53 (49)	
HCM	39 (27)	31 (28)	
Pilot	26 (18)	25 (23)	
Regional health trust			
North	20 (14)	20 (18)	192 (18)
Middle	32 (22)	23 (21)	202 (19)
West	37 (26)	23 (21)	257 (25)
South-East	52 (36)	42 (39)	394 (38)
Other	4 (3)	1 (< 1)	
Prehospital seniority			
Less than 5 years	34 (23)	26 (24)	195 (19)
6 to 10 years	39 (27)	26 (24)	259 (25)
11 to 15 years	23 (16)	19 (17)	211 (20)
16 to 20 years	22 (15)	27 (25)	180 (17)
21 years or more	27 (19)	11 (10)	200 (19)

HEMS 12 = HEMS sample 2012, HEMS 16 = HEMS sample 2016, Ground 16 = ground ambulance sample 2016, EMT = Emergency medical technician. ‘Nurse EMT’ represents nurses with authorization as an EMT. ‘Nurse’ represents nurses without authorization as an EMT. HCM = HEMS crew member

The collected data clearly reflects the professional groups in the Norwegian prehospital environment, and also the difference in team compositions between ground ambulance and HEMS. Respondents are evenly distributed geographically between the four healthcare regions, with a majority from the south-east region for all samples. Such even distribution is also observed for seniority in the prehospital environment, with a mean of at least ten years. A high number of the respondents worked directly with patients (97%).

### Construct validity and post hoc modification of the instrument

CFA was applied to determine model fit. For the original instrument, acceptable model fit was observed for the ground ambulance sample, while both HEMS samples fell below this. Post hoc modification was performed to obtain acceptable model fit for both HEMS samples. One item was initially observed with a relative weak factor loading (< 0.5): item A11 (Ground 16: 0.46, HEMS 16: 0.15). A few items with at least five standardized residual covariances above |2.5| were observed: item A11 (HEMS 16, Ground 16), item C3 (Ground 16), and item C4 (Ground 16). A post hoc modification, removing from the instrument items A11 (*when one area in this unit gets really busy, others help out*), C3 (*whenever pressure builds up, my manager wants us to work faster, even if it means taking shortcuts*) and C4 (*my local manager overlooks patient safety problems that happen over and over*), resulted in improved model fit for all three samples. Based on modification indices, items D2 (*staff will freely speak up if they see something that may negatively affect patient care)* and D5 (*in this unit, we discuss ways to prevent errors from happening again)* were also removed, with the result that acceptable model fit was obtained for all three samples (Table 3).

**Table 3:** Model fit measurement model

Indices	Guidance values	N < 250				Guidance values	N > 250			
		Original HSOPSC-S		Trimmed HSOPSC-S			Original HSOPSC-S		Trimmed HSOPSC-S	
		HEMS 12	HEMS 16	HEMS 12	HEMS16		Ground 16	Total 16	Ground 16	Total 16
SRMR	< .08	.065	.090	.048	.056	< .08	.047	.047	.028	.026
TLI	> .95	.89	.86	.97	.95	> .92	.92	.93	.98	.98
RMSEA	< .08	.066	.083	.036	.052	< .07	.054	.054	.031	.032
CFI	> .95	.91	.88	.98	.96	> .92	.94	.94	.98	.98

N = sample size, HEMS 12 = HEMS sample 2012, HEMS 16 = HEMS sample 2016, Ground 16 = ground ambulance sample 2016, Total 16 = combined HEMS and ground ambulance 2016 samples. Guidance values retrieved from [76]

In addition, based on the two HEMS samples and the ground sample, a configural invariance test was conducted to reveal whether CFA achieved adequate fit among these samples when they were tested together and freely without cross-group constraints. The test indicated that the resultant model achieved good fit (SRMR: 0.048, TLI: 0.98, RMSEA: 0.020 and CFI: 0.98), and conclusively, that configural invariance was adequate among the samples.

The factor loading values for the items in the adjusted instrument were in the range 0.56 to 0.92 (Table 4). None of the dimensions had more than one value below 0.6 or above 0.9 for any of the samples. Following the reasoning that the values should preferably be between these two values, this indicated an overall acceptable convergent validity.

**Table 4:** HSOPSC-S dimensions and items (the selected items have same wording as in the full PreHSOPSC [28])

Dimension / Item		Factor loadings			
		HEMS 12	HEMS 16	Ground 16	Total 16
Management support for patient safety					
H1	Hospital management provides a work climate that promotes patient safety.	.86	.77	.82	.82
H8	The actions of hospital management show that patient safety is a top priority.	.73	.92	.80	.80
Manager expectations & actions promoting patient safety					
C1	My manager says a good word when he/she sees a job done according to established patient safety procedures.	.85	.79	.85	.84
C2	My manager seriously considers staff suggestions for improving patient safety.	.91	.85	.90	.91
Teamwork within units					
A1	People support one another in this local unit.	.76	.84	.83	.83
A3	When a lot of work needs to be done quickly, we work together as a team to get the work done.	.77	.72	.71	.72
A4	In this local unit, people treat each other with respect.	.65	.92	.81	.83
Learning, feedback and improvement within units					
D1	We are given feedback about changes put into place based on event reports.	.67	.71	.65	.68
D3	We are informed about errors that happen in this local unit.	.80	.89	.74	.77
D4	Staff feel free to question the decisions or actions of those with more authority.	.71	.72	.70	.72
Stop working in dangerous situations					
A19	I ask my colleagues to stop work when I think the job is being done in a risky manner.	.65	.62	.66	.66
A20	I report dangerous situations when I see them.	.59	.81	.75	.76
B2	I stop working if I think it could be dangerous for me or others to continue.	.69	.68	.56	.57
Transitions and handoffs					
H3	Things “fall between the cracks”* when transferring patients from one unit to another. (*For example, patient information is not transmitted, unclear responsibility for tasks and procedures in patient handover.)	.71	.72	.66	.66
H5	Important patient care information is often lost during shift changes.	.75	.86	.74	.75
H7	Problems often occur in the exchange of information across units in the prehospital chain.	.66	.76	.69	.70
H11	Patient handovers are problematic for patients in the prehospital chain.	.61	.76	.63	.64

Dimensions and items based on original Norwegian and English versions of HSOPSC [27, 29, 32]. HEMS 12 = HEMS sample 2012, HEMS 16 = HEMS sample 2016, Ground 16 = ground ambulance sample 2016, Total 16 = combined HEMS and ground ambulance 2016 samples

The intercorrelations between the safety dimensions ranged from little/fair (0.24) to moderate (0.59) degree of relationship for the different samples (Tables 5 and 6); hence, no strong correlation (> 0.75) was observed between dimensions. One correlation between “*management support for patient safety*” and “*stop working in dangerous situations*” was observed as not significant in the HEMS 16 sample.

**Table 5:** Intercorrelation (Spearman’s Rho) of dimensions for the HEMS samples

	HEMS 12					HEMS 16				
Dimension	1	2	3	4	5	1	2	3	4	5
1. Management support for patient safety										
2. Manager expectations & actions promoting patient safety	.39					.39				
3. Teamwork within units	.48	.51				.26	.42			
4. Learning, feedback and improvement within units	.46	.41	.45			.31	.53	.28		
5. Stop working in dangerous situations	.39	.38	.42	.42		n.s.	.39	.25	.57	
6. Transitions and handoffs	.41	.29	.29	.34	.31	.52	.43	.29	.40	.40

Numbered correlations significant at the 0.01 level (2-tailed), n.s.: not significant; 0.0–0.25: little or no relationship; 0.25–0.50: fair degree of relationship; 0.50–0.75: moderate to good relationship; > 0.75: very good to excellent relationship [85]. HEMS 12 = HEMS sample 2012, HEMS 16 = HEMS sample 2016

**Table 6:** Intercorrelation (Spearman’s Rho) of dimensions for the ground ambulance and the total 2016 sample

	Ground 16					Total 16				
Dimension	1	2	3	4	5	1	2	3	4	5
1. Management support for patient safety										
2. Manager expectations & actions promoting patient safety	.46					.45				
3. Teamwork within units	.37	.43				.36	.45			
4. Learning, feedback and improvement within units	.53	.58	.40			.50	.59	.44		
5. Stop working in dangerous situations	.28	.29	.32	.30		.26	.31	.32	.34	
6. Transitions and handoffs	.38	.29	.31	.30	.24	.39	.30	.30	.31	.25

All correlations significant at the 0.01 level (2-tailed). 0.0–0.25: little or no relationship; 0.25–0.50: fair degree of relationship; 0.50–0.75: moderate to good relationship; > 0.75: very good to excellent relationship [85]. Ground 16 = ground ambulance sample 2016, Total 16 = combined HEMS and ground ambulance 2016 samples

MANOVA was conducted based on satisfactory data quality meeting different assumptions (normality, equality of variance, univariate outliers, and equality of covariance matrices). By utilizing MANOVA, Wilk’s Lambda was measured for all different employee characteristics (Table 7). Although not significant for all characteristics, overall, along with intercorrelations, acceptable discriminant validity is observed.

**Table 7:** Wilk’s Lambda

Employee characteristics	HEMS 12	HEMS 16	Ground 16	Total 16
Geographic location	.123	.048*	.001***	.001***
Competence group	.001***	.009**	.001***	.001***
Work hours	.005**	.749	.001***	.001***
Seniority in position	.814	.064	.108	.251
Seniority prehospital	.750	.578	.001***	.001***

* *p* < 0.05; ** *p* < 0.01; *** *p* < 0.001. HEMS 12 = HEMS sample 2012, HEMS 16 = HEMS sample 2016, Ground 16 = ground ambulance sample 2016, Total 16 = combined HEMS and ground ambulance 2016 samples

### Descriptive statistics and internal consistency

The mean statistics and standard deviation (SD) are presented in Table 8 for each of the measurement concepts of the modified instrument, for each of the sample groups. The patient safety climate dimensions with highest scores are “*teamwork within units*” and “*manager expectations & actions promoting patient safety”*. Overall, variance of items was considered adequate. Cronbach’s alpha coefficients varied from 0.68 (*stop working in dangerous situations*) to 0.87 (*manager expectations & actions promoting patient safety*).

**Table 8:** Means, standard deviations (SD) and Cronbach’s alpha coefficients

	HEMS 12		HEMS 16		Ground 16		Total 16	
Measurement concepts	Mean (SD)	Alpha	Mean (SD)	Alpha	Mean (SD)	Alpha	Mean (SD)	Alpha
Top level								
Management support for patient safety	3.37 (.84)	.77	3.08 (.87)	.83	3.04 (.86)	.79	3.05 (.86)	.79
Unit level								
Manager expectations & actions promoting patient safety	3.95 (.78)	.87	3.98 (.74)	.81	3.51 (.98)	.87	3.56 (.97)	.87
Teamwork within units	4.22 (.63)	.77	4.58 (.60)	.86	4.07 (.70)	.82	4.12 (.71)	.83
Learning, feedback and improvement within units	3.67 (.76)	.77	3.89 (.77)	.79	3.07 (.80)	.74	3.15 (.83)	.76
Individual behavior								
Stop working in dangerous situations	4.11 (.57)	.68	4.25 (.55)	.74	4.08 (.58)	.69	4.10 (.58)	.70
Outcome dimension								
Transitions and handoffs	3.32 (.65)	.78	3.48 (.66)	.85	3.39 (.66)	.77	3.40 (.66)	.78

HEMS 12 = HEMS sample 2012, HEMS 16 = HEMS sample 2016, Ground 16 = ground ambulance sample 2016, Total 16 = combined HEMS and ground ambulance 2016 samples

### Testing of structural model

SEM indicated satisfactory model fit for all samples, although slightly below the recommended values for the HEMS 2012 sample (Table 9). Following the criterion that paths should have at least two significant relationships, several non-significant paths were removed sequentially, starting with the highest probability (p). The final model fit remained relatively equal to the model fit of the original structure (Table 9).

**Table 9:** Model fit structure model

Indices	Guidance values	N < 250				Guidance values	*N* > 250			
		Original structure		Trimmed structure			Original structure		Trimmed structure	
		HEMS 12	HEMS 16	HEMS 12	HEMS 16		Ground 16	Total 16	Ground 16	Total 16
SRMR	< .08	.070	.060	.071	.062	< .08	.044	.040	.050	.046
TLI	> .95	.94	.96	.94	.96	> .92	.96	.97	.96	.96
RMSEA	< .08	.053	.051	.054	.048	< .07	.043	.040	.045	.042
CFI	> .95	.95	.97	.95	.97	> .92	.97	.97	.96	.97

N = sample size. HEMS 12 = HEMS sample 2012, HEMS 16 = HEMS sample 2016, Ground 16 = ground ambulance sample 2016, Total 16 = combined HEMS and ground ambulance 2016 samples. Guidance values retrieved from [76]

**Fig. 3:**
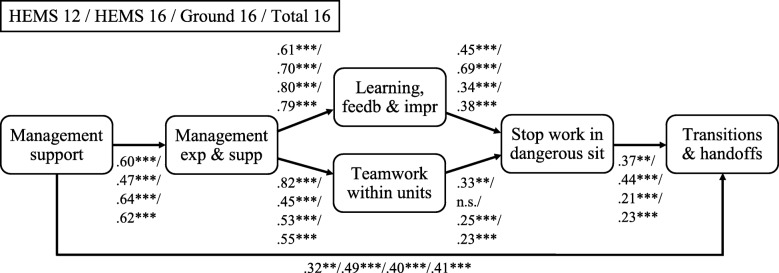
Standardized path coefficient. Note: HEMS 12 = HEMS sample 2012, HEMS 16 = HEMS sample 2016, Ground 16 = ground ambulance sample 2016, Total 16 = combined HEMS and ground ambulance 2016 samples. Management support = “*Management support for patient safety*”, Management exp. & supp = “*Manager expectations & actions promoting patient safety*”, Learning, feedb & impr = “*Learning, feedback & improvement within units*”, Stop working in dangerous sit = “*Stop working in dangerous situations*”. n.s. = not significant, **p < .01, ***p < .001

The final structure model with its standardized path coefficients is presented in Fig. 3.

After the non-significant path removal process, only one relationship remained non-significant. Surprisingly, despite our hypotheses on the safety climate in the prehospital environment, none of the dimensions “*manager expectations & actions promoting patient safety*”, “*teamwork within units*” and “*learning, feedback and improvement within units*” had two or more significant relationships on the outcome dimension “*transitions and handoffs*”. The path between “*learning, feedback and improvement within units*” and “*teamwork within units*” was also removed.

The path from the dimension “*management support for patient safety*” to “*manager expectations & actions promoting patient safety*”, and the paths from “*manager expectations & actions promoting patient safety*” to both of the unit dimensions “*teamwork within units*” and “*learning, feedback and improvement within units*” are all observed to have relatively high coefficients for all samples. This supports the importance of management levels influencing the other safety climate concepts at both team and individual levels.

A post hoc test was conducted to compare the final structural model with a model where all latent factor were specified to have direct effects on the outcome dimension “*transitions and handoffs*”. This alternative model indicated a good model fit (e.g. for the total 2016 sample; SRMR: 0.026, TLI: 0.98, RMSEA: 0.032 and CFI: 0.98). However, two of the direct paths on the outcome dimension proved non-significant; “*manager expectations & actions promoting patient safety*” and “*learning, feedback and improvement within units*”. Due to non-significant paths, and as the hypothesized model is built on a theoretical framework, the final structural model is preferred over the alternative model.

## Discussion

This study produced three major findings. Firstly, the original HSOPSC-S measurement model provided overall acceptable model fit for the ground ambulance sample, implying that the original HSOPSC-S instrument is adequate for distribution among the ground ambulance. As it does not provide model fit for any of the HEMS samples, no further psychometric properties were tested on the original HSOPSC-S instrument in this article. Secondly, a trimmed version of HSOPSC-S was observed with overall acceptable psychometric properties for the measurement model for both the ground ambulance sample and the two HEMS samples, i.e. acceptable internal consistencies and construct validity. Thirdly, by testing the theoretical, structured framework, we could observe the dynamic relation between the management and unit levels, which culminates in a significant influence on the outcome dimension “*transitions and handoffs*”. This is beneficial for an improved understanding of the prehospital patient safety climate. The second and third aforementioned findings indicate that the final model, the PreHSOPSC-S, is appropriate for generalized application in the prehospital environment.

### Modified HSOPSC-S

By performing a post hoc modification of HSOPSC-S, the results demonstrated a clearly better model fit for all samples in the measurement model. In addition, after removal of weak items, the results also indicated acceptable convergent and discriminant validity. It has been advocated a conservative approach when performing modifications, i.e. few modifications and clear interpretability [81]. The removal of five items may be argued to be a moderate modification relative to the original 22 items.

A recent article demonstrates that items A11 (*when one area in this unit gets really busy, others help out*) and C3 (*whenever pressure builds up, my manager wants us to work faster, even if it means taking shortcuts*) is challenging due to respectively not reflecting work processes and due to use of idioms, and should thus be considered candidates for removal [28]. For items C4 (*my local manager overlooks patient safety problems that happen over and over*), D2 (*staff will freely speak up if they see something that may negatively affect patient care)* and D5 (*in this unit, we discuss ways to prevent errors from happening again),* it may be argued that these items possibly reflect variance in local practices which are not sufficiently coherent within the prehospital environment. Such variance may stem from the lack of common explicit knowledge (procedures, routines etc.) or tacit knowledge (norms, common understanding etc.). In a hospital, it is reasonable that local practices (unit level) is more aligned with regional (hospital level). However, due to the scattered nature of the prehospital domain this is not granted as such. As the argument has not been sufficiently demonstrated, it may constitute a hypothesis for future research.

Normally, modification may benefit on random variation in an obtained sample and should be viewed as tentative until cross-validated with an independent sample [81]. In this study, the modification was performed with its basis in three independent samples, either within the same work area with a split of four years (the two HEMS samples) or in different work areas (the ground ambulance and HEMS samples). After modification of the original measurement model and the structural framework model, the CFA demonstrated acceptable model fit for all samples against the recommended values. The result of the post hoc modification is the PreHSOPSC-S, which is a shorter – but stronger – instrument suitable for application in a broader prehospital environment.

As a shorter instrument, the PreHSOPSC-S may be a significant contribution to performing safety climate assessments in the prehospital environment. The prehospital environment is, relative to hospitals, geographically scattered, and digital surveys are easier to distribute than paper surveys. An issue with digital surveys is that they may generate a relatively lower response rate [86], and a shorter version may increase the response rate and representativeness. In addition, the practical administering and analysis of a shorter survey makes it easier to perform more frequent measuring. It has been demonstrated that received feedback improves safety climate [87], which implies that providing feedback based on the measurement results to the EMS providers may also result in improved safety climate. An effective approach for more frequent measuring is to attach the survey to existing frequently performed measuring tools such as employee surveys or re-certification tests.

The original full version of the Norwegian HSOPSC comprises 13 safety climate dimensions, consisting of 46 items, in addition to two single-item outcome items [29, 35], and a relevant question is whether the PreHSOPSC-S is to be preferred over the original full HSOPSC. In study design, it has become more important to consider specific measurement instruments, level of analysis and the selection of outcome measures [88]. Whether the original or the short version should be applied for measurements depends on the objectives. If efficiency, speed and frequent measurement constitute the goal, a shorter version may be preferred. On the other hand, if an organization is conducting comprehensive improvement programs, and there is a desire to measure changes in the patient safety climate, a full version covering all dimensions may be preferred. In essence, different objectives require different measuring instruments, implying that several validated instruments with different perspectives and outcomes are beneficial for both assessments of and research on patient safety climate in the prehospital domain.

### Theoretical and practical implications

**Fig. 4:**
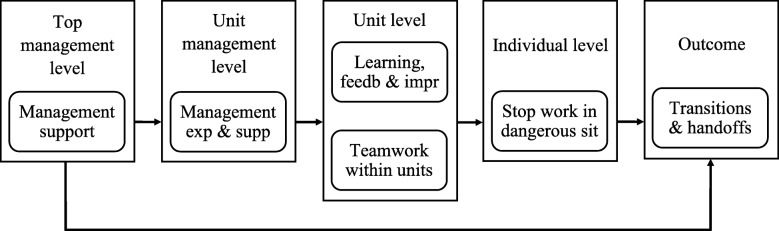
Structured framework for the prehospital environment. Note: Management support = “*Management support for patient safety*”, Management exp. & supp = “*Manager expectations & actions promoting patient safety*”, Learning, feedb & impr = “*Learning, feedback & improvement within units*”, Stop working in dangerous sit = “*Stop working in dangerous situations*”

A structural model is valuable for understanding the multilevel relationships in the prehospital domain. The structured framework for the prehospital environment based on our results is illustrated in Fig. 4.

The final model was observed with relational paths from top management via unit management and unit level to the individual level, supporting the basic principle of multilevel safety climate [38]. This observed hierarchical relationship between levels implies that the change of patient safety climate on one level would influence subordinate levels, ultimately affecting safety outcomes. All samples supported a strong positive influence from top management on unit management, and from unit management on the unit level. Based on the theoretical framework on which HSOPSC-S is based, several strategies may be chosen for how to use the structural components in improving safety in organizational settings [29]. An example of such a strategy is to define and implement a multilevel safety program, whose goal is to improve safety culture and safety behavior [89]. Top management may put patient safety on the agenda, integrating it into policies and agendas, ensuring that unit management adopts these into practice. Another strategy is leadership development initiatives to improve the safety climate [90], involving leaders on all levels.

#### Top management level influence on transitions & handoffs

The direct path from the top management level dimension *“management support to patient safety*” on the outcome dimension “*transitions and handoffs*” may be related to the EMS providers’ perception of the overall healthcare system. This implies that top management has an important direct role in patient safety in transitions and handoffs, through the quality of internal work processes, procedures and routines for patient transitions and handoffs in the prehospital domain. Patient transitions and handoffs are also organizational interfaces [91]. When commencing transitions and handoffs between the prehospital and hospital domains, EMS workers may perceive this as an interface, where both these cultural domains influence the outcome of patient transitions and handoffs. This may be seen in light of patient transitions and handoffs being considered an important area of contact between employees and the hospital [23]. An implication of this is that the better the EMS providers perceive the management support to patient safety, the better they perceive the hospital management effort to provide and maintain effective and safe systems for transitions and handoffs. In essence, top management’s expressed values and priorities are important for obtaining improved outcomes.

#### Unit level influence on transitions & handoffs

The lack of a direct path from the outcome dimension “*transitions and handoffs*” to both of the unit levels “*teamwork within units*” and “*learning, feedback and improvement within units*”, demonstrates a full mediation by individual safety behavior. An implication of this finding is that workers’ perception of the safety climate within the unit differs from their perception of the safety climate in transition and handoff situations. This observation was surprising, as safe transitions and handoffs have been linked to interdisciplinary team effort and team communication skills [69]. The explanation may be that transitions and handoffs often involve collaboration across units in the prehospital chain, which implies that the safety climate in this broader domain is different from the safety climate related to teamwork within the unit.

Another explanation may be related to the fact that two of the three samples applied in this study are retrieved from HEMS workers. In the ground ambulance, tasks related to patient diagnostics, treatment, transitions and handoffs are often shared by the team. The composition of a HEMS team has a higher degree of individual professionalism than that of other prehospital team compositions, with each professional having their defined set of tasks and areas for which they are responsible [30]. The physician in HEMS is mainly responsible for patient diagnostics and treatment, in addition to transitions and handoffs, i.e. transfer of patient information, responsibility and accountability. However, patient safety processes and climate within the unit influence the individual behavior, hence having an indirect influence on the outcome of transitions and handoffs. This implies the importance of the continued focus on teamwork and the focus on learning, feedback and improvement within the unit.

#### Individual level influence on transitions & handoffs

The dimension “*stop working in dangerous situations*” is a particular type of safety behavior [29], and in practice such behavior is likely to be primarily related to high-risk tasks in the prehospital domain such as driving [92] or clinical judgement and decision-making [2, 93]. An example of “stop working” behavior is cross-monitoring, i.e. the possibility to ask critical questions or voice concerns if one believes that an action may harm the patient [56, 63]. The direct path from the safety behavior dimension “*stop working in dangerous situations*” to the outcome dimension “*transitions and handoffs*” implies that, if individuals perceive that they can “stop working” when performing safety-related tasks, it also increases quality and safety in the individual tasks related to patient transitions and handoffs. Implementing different “stop working” techniques in training programs, simultaneously with promoting and cultivating such behavior, is likely to improve both the outcome of transitions and handoffs, as well as overall patient safety.

### Limitations and future research

The response rate for the ground ambulance sample was low relative to other similar studies [32–34, 36, 94]. This may induce non-response bias, meaning that the group of non-responders differs from the group of responders. See [28] for an extended discussion on possible reasons for the low response for the sample retrieved in 2016. The two HEMS samples had higher response rates, but sample sizes are relatively small, thus increasing the likelihood for Type II errors. However, being able to test on all these three different samples outweighs the overall likelihood for Type II errors.

A disadvantage of a modified instrument is the lack of opportunity to compare with other studies undertaken. Although the PreHSOPSC-S is tested for a major part of the prehospital environment, further research should test and validate the instruments (both original and modified) for other parts, to obtain a more generalized instrument for measuring safety climate. Use of the instrument in a new context is a limitation in itself, and development of a new instrument targeted on an prehospital context may be a better solution. However, we have demonstrated both a multilevel structural relationship and a relational effect from safety climate dimensions on patient transitions and handoffs, which may be included when designing new instruments.

PreHSOPSC-S is based on self-reported data and has not been tested for predictive validity, i.e. not compared with an external criterion [75], such as degree of error reporting, degree of patient compensation due to harm, or effect of intervention. Before such testing against external criteria is undertaken, the impact on the prehospital safety climate is not fully known.

## Conclusion

Researching and measuring safety climate may provide a valuable input in responding to potential patient safety issues in the prehospital domain. Normally, relative few dimensions are addressed at a time when performing actions to improve the safety climate [15]. Through use of a multilevel approach, safety programs may be designed to include several levels and safety climate dimensions, simultaneously. The literature suggests to start with surveys when initiating the process of measuring the current safety climate status [95, 96]. Hence, it is satisfying to provide a validated instrument for measuring the patient safety climate with a multilevel perspective in a broader prehospital context: the PreHSOPSC-S. The validity for use throughout the prehospital domain is utterly demonstrated by the uniqueness of the samples, as the HEMS differs substantially from the ground ambulance, and the two HEMS samples were retrieved at two points in time. This study also demonstrated that the multilevel patient safety climate is positively related to the outcome dimension related to patient transitions and handoffs. To our knowledge, this is the first prehospital patient safety climate study with a multilevel perspective and with the use of transitions and handoffs as an outcome. In the prehospital domain, safe patient transitions and handoffs are considered important, and a structured framework will constitute an important contribution to prehospital safety climate assessment and research.

## Data Availability

The data sets generated and analyzed during the current study are not publicly available, as further papers will be written based on the data sets; however, they are available from the corresponding author on reasonable request.

## Abbreviations

AHRQ: Agency for Healthcare Research and Quality
CFA: Confirmatory factor analysis
CFI: Comparative fit index
EFA: Exploratory factor analysis
EMS: Emergency medical services
EMT: Emergency medical technician
FW: Fixed wing
HCM: HEMS crew member
HEMS: Helicopter emergency medical services
HSE: Health, safety and environment
HSOPSC: Hospital Survey on Patient Safety Culture
MANOVA: Multivariate analysis of variance
PreHSOPSC: Prehospital Survey on Patient Safety Culture
RMSEA: Root mean square error of approximation
SAR: Search and rescue services
SD: Standard deviation
SEM: Structured equational modelling
SRMR: Standardized root mean square residual
TLI: Tucker-Lewis index
